# IL-17 Expression in the Time Course of Acute Anti-Thy1 Glomerulonephritis

**DOI:** 10.1371/journal.pone.0156480

**Published:** 2016-05-31

**Authors:** Tanja Loof, Stephanie Krämer, Jens Gaedeke, Hans-Hellmut Neumayer, Harm Peters

**Affiliations:** 1 Department of Nephrology and Center of Cardiovascular Research, Campus Charité Mitte, Charité–Universitätsmedizin Berlin, Berlin, Germany; 2 German Institute of Human Nutrition, Potsdam-Rehbrücke, Germany; University of Louisville, UNITED STATES

## Abstract

**Background:**

Interleukin-17 (IL-17) is a new pro-inflammatory cytokine involved in immune response and inflammatory disease. The main source of IL-17 is a subset of CD4^+^ T-helper cells, but is also secreted by non-immune cells. The present study analyzes expression of IL-17 in the time course of acute anti-thy1 glomerulonephritis and the role of IL-17 as a potential link between inflammation and fibrosis.

**Methods:**

Anti-thy1 glomerulonephritis was induced into male Wistar rats by OX-7 antibody injection. After that, samples were taken on days 1, 5, 10 (matrix expansion phase), 15 and 20 (resolution phase). PBS-injected animals served as controls. Proteinuria and histological matrixes score served as the main markers for disease severity. In *in vitro* experiments, NRK-52E cells were used. For cytokine expressions, mRNA and protein levels were analyzed by utilizing RT-PCR, *in situ* hybridization and immunofluorescence.

**Results:**

Highest IL-17 mRNA-expression (6.50-fold vs. con; p<0.05) was found on day 5 after induction of anti-thy1 glomerulonephritis along the maximum levels of proteinuria (113 ± 13 mg/d; p<0.001), histological glomerular-matrix accumulation (82%; p<0.001) and TGF-β1 (2.2-fold; p<0.05), IL-6 mRNA expression (36-fold; p<0.05). IL-17 protein expression co-localized with the endothelial cell marker PECAM in immunofluorescence. In NRK-52E cells, co-administration of TGF-β1 and IL-6 synergistically up-regulated IL-17 mRNA 4986-fold (p<0.001).

**Conclusions:**

The pro-inflammatory cytokine IL-17 is up-regulated in endothelial cells during the time course of acute anti-thy1 glomerulonephritis. In vitro, NRK-52E cells secrete IL-17 under pro-fibrotic and pro-inflammatory conditions.

## Introduction

As shown by the foundation "World Kidney Day", more than 500 million patients pose chronic kidney diseases due to various underlying immune and non-immune entities [1]. The process of glomerulonephritis formation and disease progression is complex and involves multiple interactions between resident cells, infiltrating cells, and their soluble factors. This has already been well addressed [2–4]. However, the interaction between pro-inflammatory and pro-fibrotic factors in this process is still not fully understood. In general, acute and chronic glomerular disorders are characterized by a three-step sequence of renal damage. This begins with a deterioration of resident glomerular structures (injury phase). Then, injured resident cells respond, increasing extracellular matrix production and accumulation (matrix expansion phase). Finally, the matrix accumulation may resolve (in the resolution phase) but this can also advance to subsequent nephron destruction (progression phase). Increased extracellular matrix expansion, in which the cytokine TGF-β1 represents the key mediator of matrix expansion and fibrosis, correlates with the ongoing loss of kidney function [5].

The cytokine IL-17, together with its IL-17 receptor signaling axis is strikingly different in sequence and structure from those of other cytokine families [6]. IL-17A and IL-17F, approximately 55% homologous, are the best-characterized family members of the IL-17 cytokine family. These comprise six structurally related isoforms (IL-17 A to F) [7]. Both IL-17A and IL-17F have been shown to promote the inflammatory pathology of a number of autoimmune diseases such as rheumatoid arthritis, lupus erythematodes, psoriasis, and asthma [8–11]. The main source of IL-17 is a subset of CD4+ T-helper cells known as TH17 cells [12]. IL-17-expressing TH17 cells have been known to play a role in inflammatory diseases [9–11] and in renal inflammation [13–15]. In addition γδ T-cells [16], natural killer T-cells [17], neutrophils [18–20], as well as mast cells [21] are known to express IL-17. IL-17 production is induced by a combination of IL-6 and TGF-β1, and maintained by IL-23 [22–27]. However, IL-17 is also required for host defense and acts mainly by inducing neutrophil recruitment [28–30]. With regard to the kidney, IL-17 expression has been shown in tubular epithelial cells in human antibody-mediated kidney rejection [31]. In human proximal tubular epithelial cells, in turn, IL-17 has been found to stimulate the expression of the inflammatory factors IL-6, CXCL8 and CCL2 [32].

The aim of this study was to characterize IL-17 expression over the course of acute anti-thy1 glomerulonephritis, to identify the IL-17 expressing cells, and to elucidate further the IL-17 / TGF-β1 interaction in renal cells.

## Methods

### Materials

Unless otherwise indicated, all materials and chemicals were purchased from Sigma Chemical-Aldrich (Taufkirchen, Germany).

#### Animals and induction of acute anti-thy1 glomerulonephritis

All animal experiments were conducted in accordance with good animal practice as defined by FELASA (www.felasa.eu/guidelines.php) and the national animal welfare body GV-SOLAS (www.gv-solas.de/index.html). The experiments were approved by our local governmental animal welfare authority (Landesamt für Gesundheit und Soziales, Berlin, permit number: G0144/06).

Male Wistar rats were obtained from Charles River (Sulzfeld, Germany), fed a normal protein diet (22% protein; Altromin, Lage, Germany) and housed in a constant-temperature room with a 12:12-h dark-light cycle. Acute anti-thy1 glomerulonephritis was induced as previously described. Briefly, monoclonal OX-7 antibody (1 mg/kg body weight in PBS) was injected to induce a complement and NO-dependent lysis of mesangial cells [33]. Animal specimens were obtained on days 1, 5, 10, 15 and 20 after disease induction (n = 6 for each group: 24h, d5, d10, d15, d20). PBS-injected animals served as controls (con, n = 6).

#### Urine collection and measurement of renal function

Animals were housed in individual metabolic cages (Techniplast, Buguggiate, Italy) for urine collection 24 h before the end of experiment. Urinary protein was measured by using the pyrogallol red technique as previously described [34]. Proteinuria is expressed as milligrams of protein per 24 h.

#### Histological examinations

Renal cortical tissue was fixed for 2.5 h in Carnoy´s solution (acetic acid—methanol—chloroform 10:60:30), followed by a fixation for 12 h in 100% ethanol and 1 h in xylol. All histological analyses were performed in a blinded fashion. 3-μm sections of paraffin embedded tissues were stained by using the periodic acid-Schiff (PAS) stain. Glomerular fibrosis was calculated by a computer-based histomorphometric analysis involving a Zeiss Axio Imager A.1 light microscope, a PL-A662 video camera, and the Axiovision 4.1 image-analysis system [34]. The level of glomerular matrix expansion was analyzed by calculating the relative degree of mesangial-matrix-occupying area (in percent) of 15 glomeruli from every rat [35].

For *in situ* hybridization, 4% formalin-fixed tissue samples were embedded in paraffin and cut into 3-μm sections, dewaxed, rehydrated, incubated with proteinase K (Dako, Glostrup, Denmark), rinsed twice in PBS, and then post-fixed in 4% (w/v) paraformaldehyde for 5 min. Antisense RNA probes were prepared from cDNA and labeled with digoxigenin (DIG) by using a DIG RNA labeling kit (Roche, Mannheim, Germany). After washing in PBS and incubation for 10 min in 0.1 M triethanol acetic anhydride pH 8, the sections were again dehydrated and hybridized at 37°C overnight with a DIG-labeled RNA probe specific to rat IL-17A in 50% formamide, 10 μg/ml sheared salmon sperm DNA (Ambion, Kaufungen, Germany), 5x Denhardt´s solution, 5x SCC, 0.1% SDS, and 5 μg/ml heparin. Subsequently, tissues were washed for 30 min three times with 4x SCC at 37°C, and then for 20 min twice with 0.1x SCC at room temperature and finally once with PBS. Hybridized DIG was detected using an anti-DIG/HRP-labeled antibody (Dako, Glostrup, Denmark) at a concentration or 1:2000 for 2 h at 37°C followed by reaction with the SigmaFast^™^ BCIP/NBT- HRP substrate according to the manufacture's protocol.

For immunofluorescence analysis a cold-light source (HAL 100FluoArc, Carl Zeiss Vision, Munich, Germany) was employed, and detection of IL-17 and TGF-β1 was carried out on 3-μm sections of Carnoy's fixed kidney tissue. Sections were rehydrated followed by two min of pressure cooking with EDTA-buffer (10 mM EDTA, 0.05% Tween 20, pH 9.0). After blocking by 20% BSA (in aqua dest), tissues were incubated for 1 h with specific goat anti-rat IL-17 1:200, rabbit anti-rat TGF-β1 1:200 and goat anti- rat PECAM-1 antibody at 1:200 (all Santa Cruz, Heidelberg, Germany) or rabbit anti-rat Synaptopodin also at 1:200 (abcam). Subsequently, slides were washed using 1x TBS (three times for 5 min each) and incubated with the secondary fluorochrome-linked antibody Alexa Fluor 488 or 568 (Invitrogen, Eugene, Oregon, USA) for 30 min. Prior to nuclear staining with 4´-6´-diamidino-2´-phenylindol (DAPI), (Invitrogen, Eugene, Oregon, USA) for 30 s, slides were washed 3 times for 5 min each with 1x TBS. Finally, tissue sections were covered with Fluoromount-G (Southern Biotech, Birmingham, USA) and stored at -20°C until examination. For a list of the primary antibodies used, see Table 1.

**Table 1:** Antibodies used for immunohistochemistry and immunofluorescence.

Antigen	Manufacturer	Host	Isotype	Catalog No.
IL-17	Santa Cruz Biotechnology, Inc., CA, USA	goat	IgG	SC6077
TGF-β1	Santa Cruz Biotechnology, Inc., CA, USA	rabbit	IgG	SC146
PCAM-1	Santa Cruz Biotechnology, Inc., CA, USA	goat	IgG	SC17019
Synaptopodin	Abcam, MA, USA	rabbit	IgG	ab101883
DIG	DAKO, Glostrup, Denmark	rabbit	IgG	P5104

#### RNA preparation and RT-qPCR

The gene expression level of IL-17, TGF-β1, IL-6 and GAPDH/ β-actin was quantitated by Q-PCR as previously reported. Briefly, total RNA were isolated by using the Trizol^™^ reagent (Invitrogen, Darmstadt, Germany) according to the manufacturer’s instructions. After isolation from each sample, 1 μg of total mRNA was used for cDNA synthesis by reverse transcription using the RNA PCR Core kit (Roche, Applied Biosystems, New Jersey, USA). Predesigned primers specific for the target were purchased from TIB MOLBIOL (Berlin, Germany). Each reaction consisted of 10 μl containing 2x Master Mix (quantifast SYBR Green PCR kit; Qiagen, Hilden, Germany), primer and cDNA. Amplification conditions consisted of 50 cycles at 95°C for 10 s, annealing 10 s (for list of temperature, see Table 2), and incubation at 72°C for 20 s. Amplification and fluorescence measurements were performed using the Mastercycler^®^ ep realplexS system (Eppendorf, Hamburg, Germany). mRNA expression was normalized using GAPDH in aGN experiments and β-actin in cell culture experiments as an endogenous control to correct for differences in the amount of total RNA originally added to each reaction. For analysis, the relative quantification ΔΔCP method was used. First, the crossing point (CP) of target gene was subtracted from the CP of the housekeeping gene. The result of this is the ΔCP value. ΔΔCP was calculated by subtraction of the ΔCP mean value of controls from the ΔCP of treated samples. The optimal PCR efficiency (E = 2) is represented by a DNA doubling at each cycle. Hence, the expression ratio of target gene to untreated control is expected to be **2**^−ΔΔ***CT***^ [36]. For a list of the primer pairs used, see Table 2.

**Table 2:** Annealing temperature and sequences of the primers.

Target	Annealing Temperature	forward 5´-3´	backward 5´-3´
β-actin	59°C	agccatgtacgtagccatcc	accctcatagatgggcacag
GAPDH	59°C	tgccactcagaagactgtgg	ttcagctctgggatgacctt
TGF-β1	61°C	gtcaactgtggagcaacacg	agacagccactcaggcgtat
IL-17	60°C	actttccgggtggagaagat	cttaggggctagcctcaggt
IL-6	60°C	accacccacaacagaccagt	cagaattgccattgcacaac

#### Cell culture experiments

The rat proximal tubular cell line NRK-52E was obtained from DSZM (DSMZ no. ACC 199, Heidelberg, Germany) and grown in DMEM supplemented with 10% fetal calf serum, 100 μg/ml penicillin, 100 μg/ml streptomycin and 2 mM L-glutamine (all from Biochrom, Berlin, Germany) in an atmosphere of 5% CO_2_ at 37°C. Cells from passages 5–10 were used. Before treatment, cells underwent 24 h of starvation for cell cycle synchronization. To further elucidate TGF-β1, IL-6 and IL-17 mRNA expression, NRK-52E cells were stimulated by a high glucose content of 25 mM (high glucose, HG) over the time periods indicated. NRK-52E cells incubated with 5 mM glucose, and 20 mM mannose served as an osmotic control. To mimic conditions in acute anti-thy1 glomerulonephritis, NRK-52E cells were co-stimulated by using 10 ng/ml of IL-6 and 5 ng/ml TGF-β1. The specific inhibitor of TGF-β superfamily activin receptor-like kinase (ALK) SB 431542 (10 μM) was used to further characterize the TGF-β1/ IL-17 network in NRK-52E cells. In all experiments, native untreated NRK-52E cells served as control.

Analysis of TGF-β1, IL-6 and IL-17 mRNA employed RT-PCR as described above. For immunofluorescence staining, NRK-52E cells under various conditions were fixed on slides (5 min in 100% ethanol at -20°C and 2 min in 100% acetone at -20°C), blocked using HEPES/ BSA-buffer (20 mM HEPES, 1% BSA in PBS) and incubated with specific goat anti-rat IL-17 (Santa Cruz, Heidelberg, Germany) and/or rabbit- anti rat TGF-β1 antibody (Santa Cruz, Heidelberg, Germany) for 60 min at 37°C, followed by four 5 min washings with HEPES-buffer (20 mM HEPES in PBS). Finally, cells were incubated with a secondary fluorochrome-conjugated antibody (AlexaFluor 568 goat anti-rabbit IgG (H+L) for TGF-β1; donkey anti-goat IgG (H+L) for IL-17), washed again and nuclear stained with DAPI for 30 s, washed 3 times for 5 min each and then covered with Fluoromount-G (Southern Biotech, Birmingham, USA).

#### Statistical analysis

Results are expressed as mean +/- standard error of the mean. For statistical comparison, *p* values were calculated with *Student´s t-test*, and *p<0*.*05* was considered as significant.

## Results

### Disease course of acute anti-thy1 glomerulonephritis

**Fig 1:**
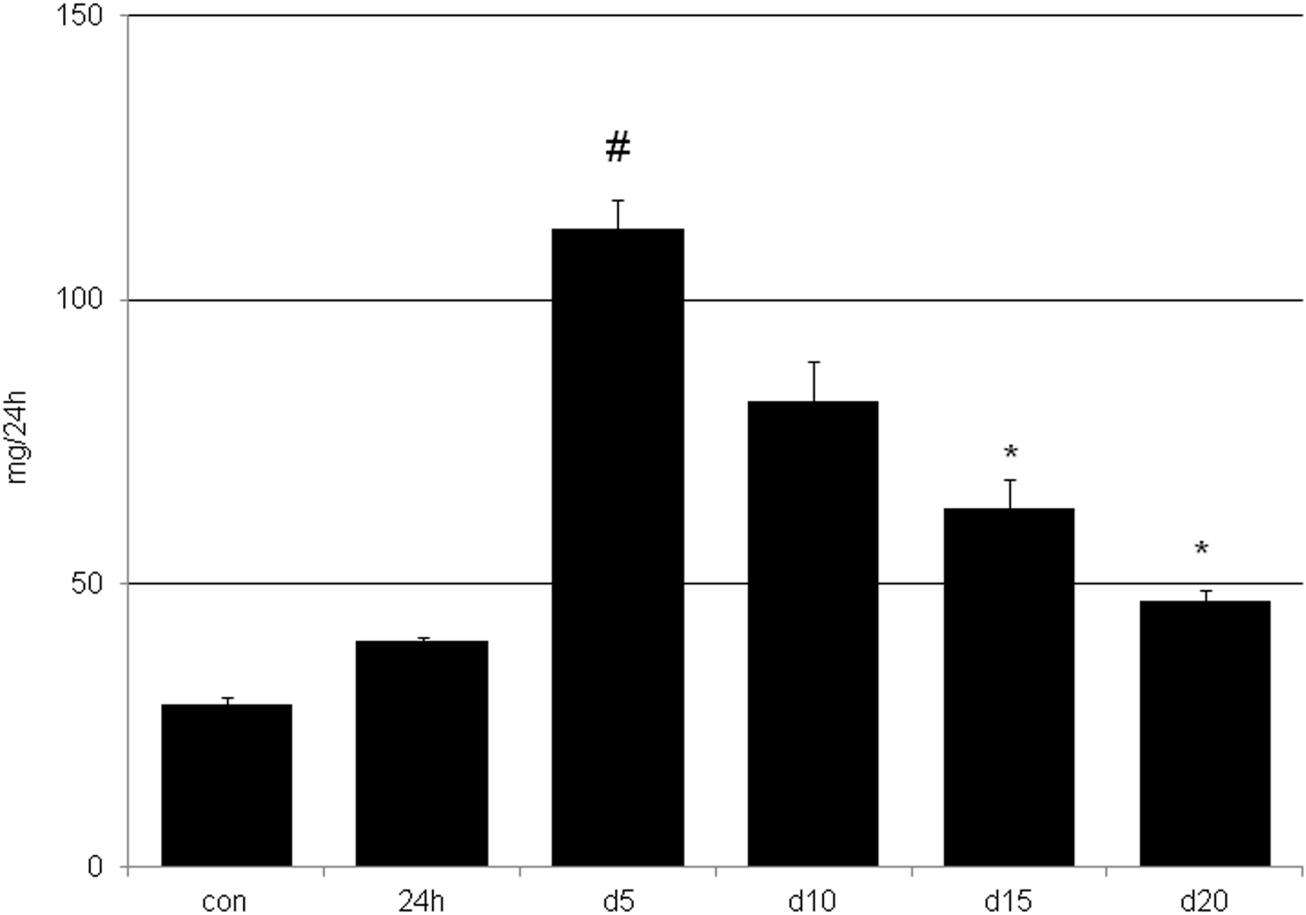
Proteinuria over the time course of acute anti-thy1 glomerulonephritis. The disease was induced by a single OX-7 antibody injection. Control animals received the same volume of PBS. Animals were housed in metabolic cages, urine was collected for 24 h and proteinuria was measured by a modified pyrogallol red method 24h = animals after 24 hours after aGN induction, d5 = animals on day 5 after aGN induction, d10 = animals on day 10 after aGN induction, d15 = animals on day 15 after aGN induction, d20 = animals on day 20 after aGN induction are shown. (# p< 0.001 vs. con; *p<0.05 vs. d5).

**Fig 2:**
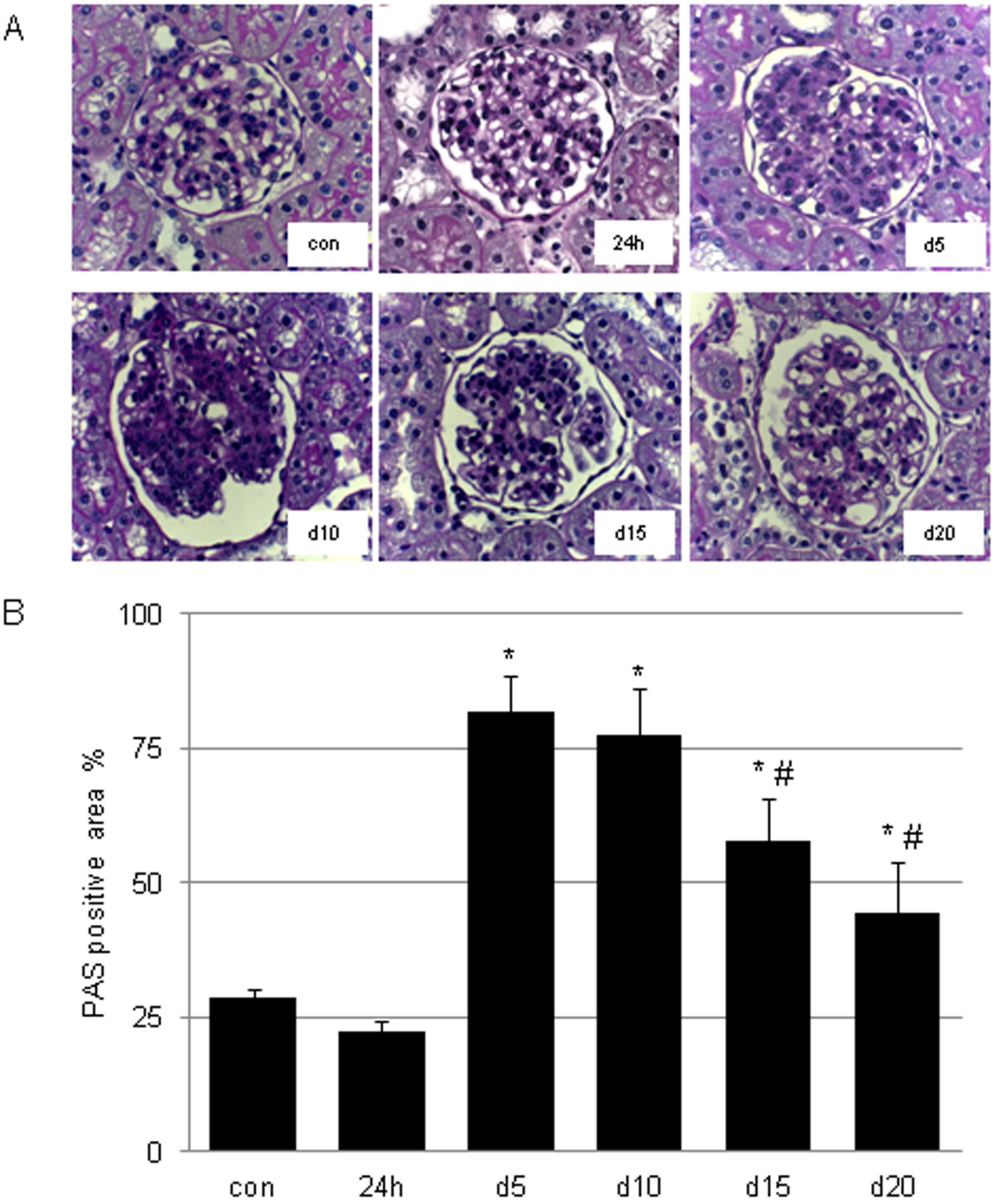
**(A) PAS-stained tissue sections over the time course of acute anti-thy1 glomerulonephritis (aGN).** Representative pictures of periodic acid-Schiff (PAS) stainings of kidneys tissue sections from con (PBS-injected control) animals, 24h = animals after 24 hours after aGN induction, d5 = animals on day 5 after aGN induction, d10 = animals on day 10 after aGN induction, d15 = animals on day 15 after aGN induction, d20 = animals on day 20 after aGN induction are shown. Magnification here is x400. **(B)** PAS-stained kidney sections were scored. (# p< 0.001 vs.d5; * p<0.001 vs. con).

24h after disease induction proteinuria decreases slightly (40mg ± 2mg/24h). Proteinuria was maximally increased five days after disease induction (113 ± 13 mg/24h vs. con 29 ± 3 mg/24h, p<0.001) and declined thereafter towards normal levels (d10 82 ± 17 mg/24h, d15 63 ± 13 mg/24h, d20 47 ± 5 mg/24h; see Fig 1). Fig 2A shows characteristic histological photographs of glomerular changes. In the matrix expansion phase a considerable histological matrix accumulation was found (d5 82 ± 7% vs. con 29 ± 2%, p<0.001; d10 78 ± 8%, p<0.001), with a decline in the subsequent matrix resolution phase (d15 58 ± 8%, p<0.001, d20 44 ± 4%, p<0.01) (Fig 2B).

### Glomerular TGF-β1, IL-6 and IL-17 mRNA expression

**Fig 3:**
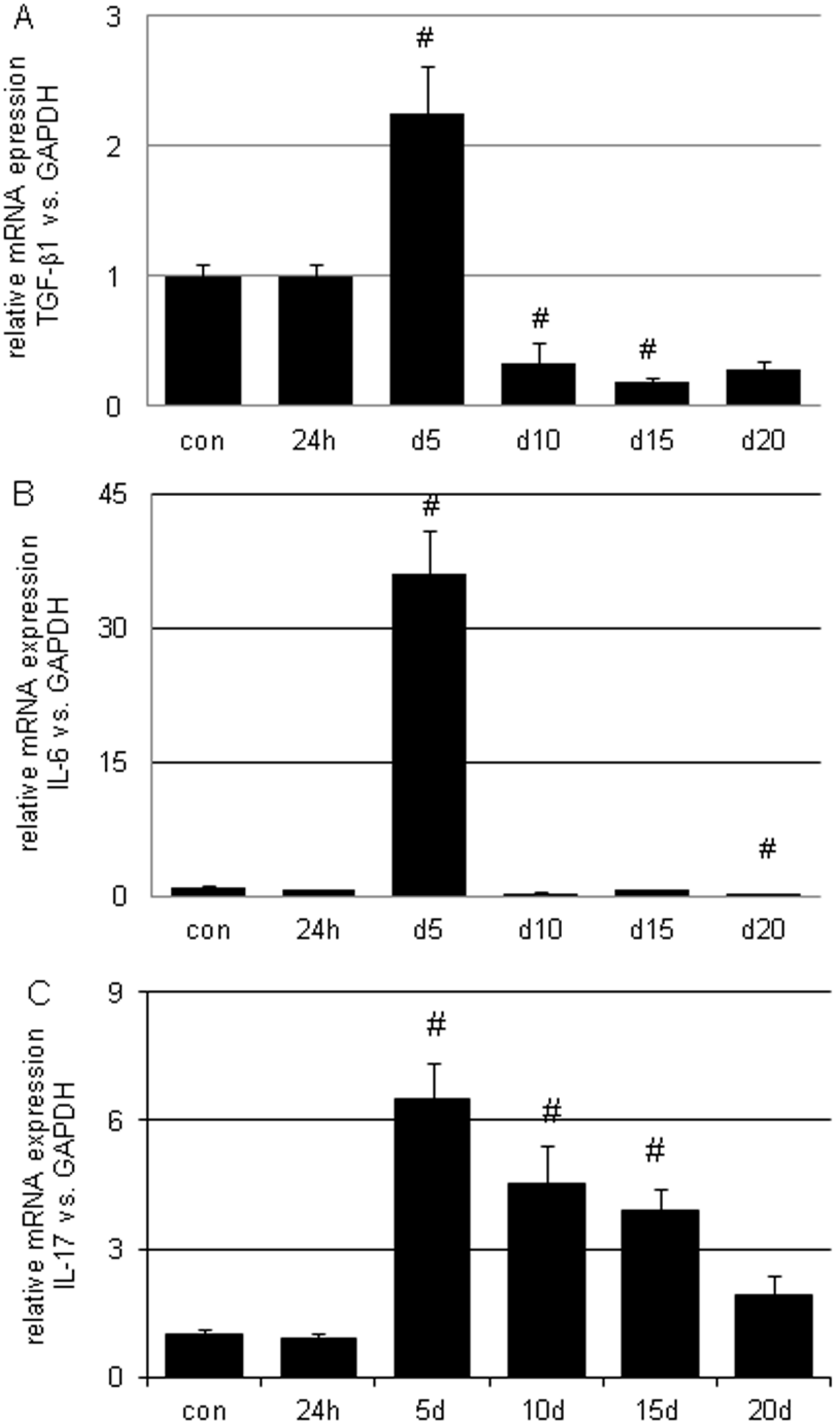
Glomerular mRNA expression of IL-6, TGF-β1 and IL-17 over the time course of acute anti-thy1 glomerulonephritis (aGN). The disease was induced by a single OX-7 antibody injection. Control animals (con) received the same volume of PBS. Animals were sacrificed, glomeruli were harvested and graded using the sieving technique and mRNA was isolated using TRIZOL reagent. After reverse transcription mRNA, expression levels were measured by real time PCR. For analysis, the relative quantification ΔΔCP method was used. (# p<0.05 vs. con).

No significantly increase in TGF-β1, IL-6 and IL-17 mRNA expression were found 24h after disease induction. IL-17 mRNA expression was significantly increased by 6.5-fold (p<0.05) on d5, which gradually decreased to 1.9-fold (p<0.05) on d20. TGF-β1 mRNA expression was maximally elevated on d5 by 2.2-fold (p<0.05), whereas it was found to be reduced to 0.33-fold on d10 (p<0.05), 0.19-fold on d15 (0.19 fold con; p<0.05) and 0.28-fold on d20 (p<0.05) when compared to normal controls. IL-6 mRNA expression was at a maximum on d5 (36-fold increase; p<0.05) and had a similarly lower expression on d10 (0.41-fold), d15 (0.71-fold) and d20 (0.25-fold; p<0.05) in comparison with the control animals (Fig 3).

### IL-17 protein expression in acute anti-thy1 glomerulonephritis

**Fig 4:**
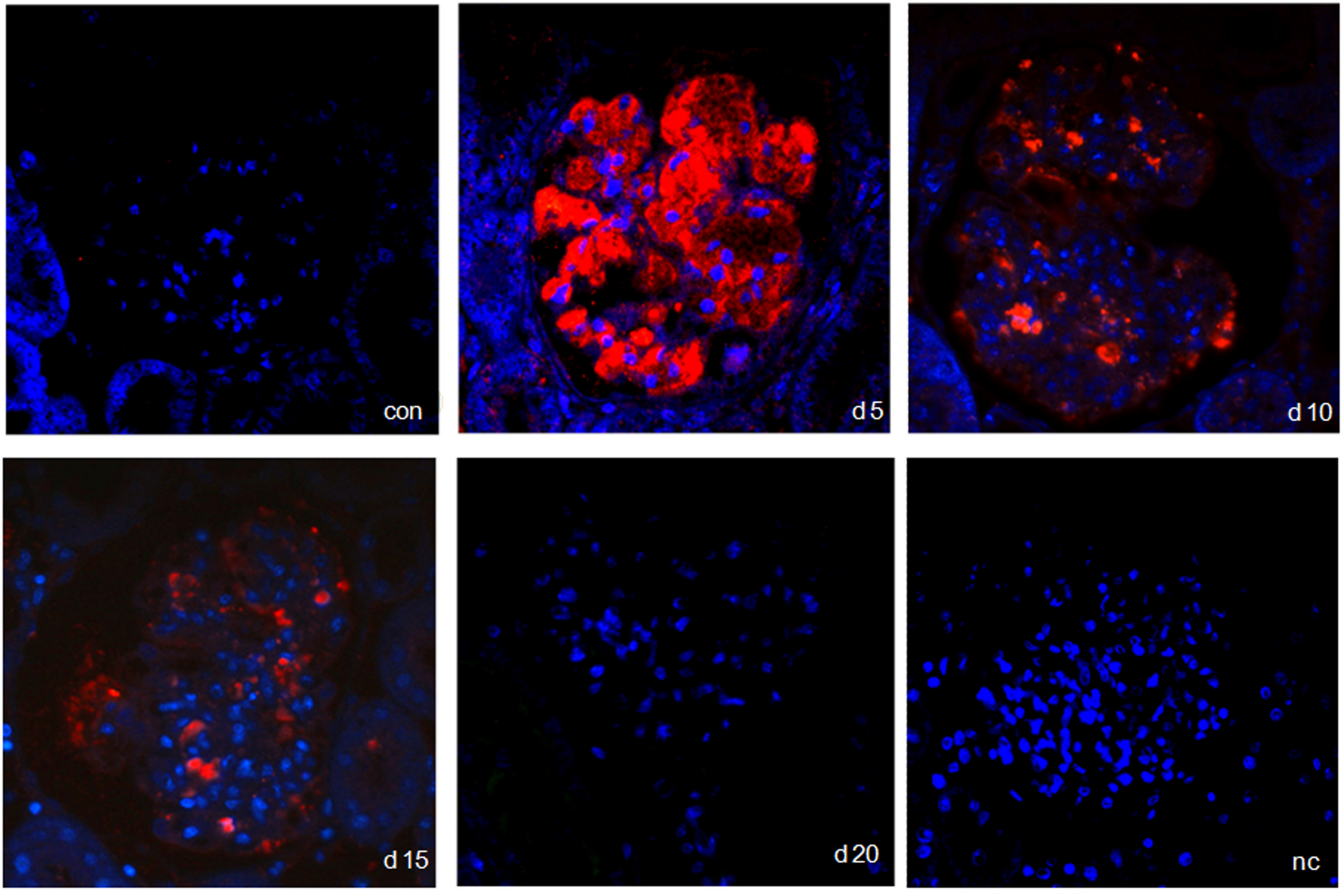
Immunostained tissue sections over the time course of acute anti-thy1 glomerulonephritis (aGN). Representative pictures showing IL-17-immunostained kidneys from con (PBS-injected control) animals, d5 = animals on d5 after aGN induction, d10 = animals on day 10 after aGN induction, d15 = animals on day 15 after aGN induction and nc = negative control are shown. IL-17 red, nucleus blue (magnification x 400).

**Fig 5:**
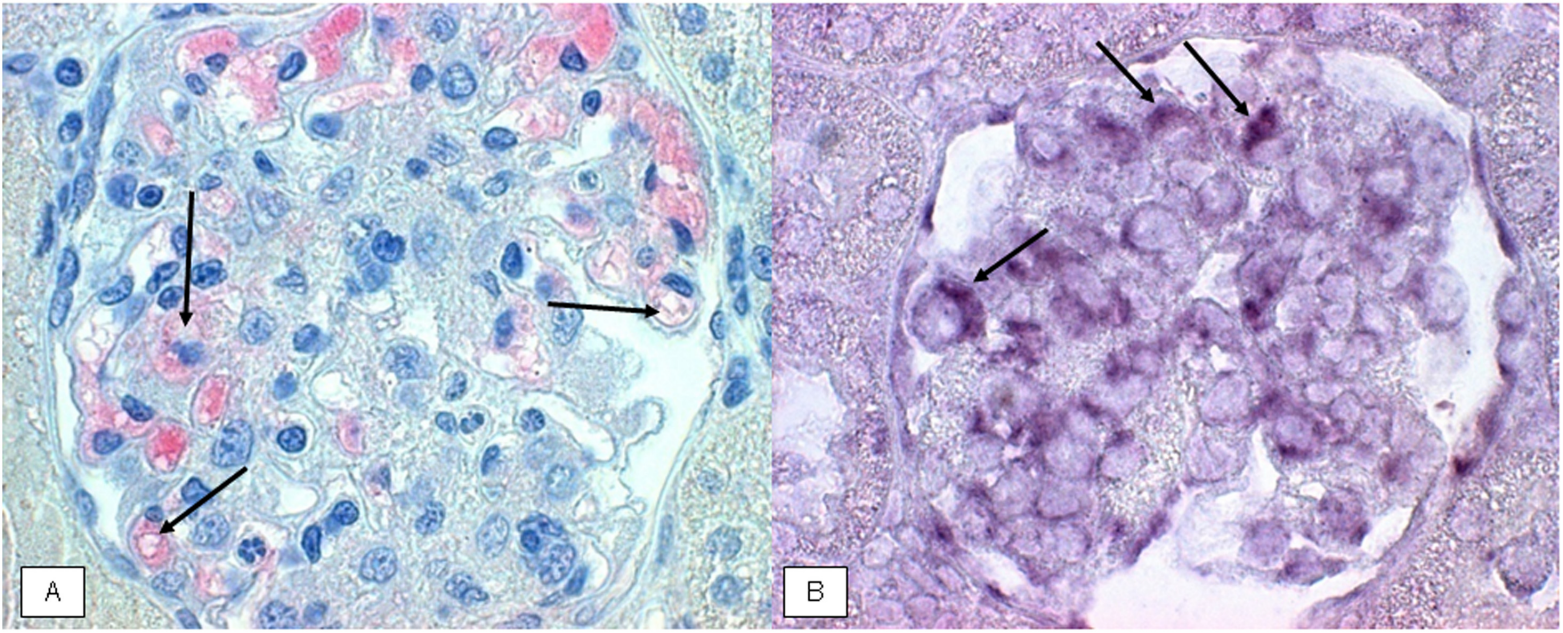
Glomerular protein expression and *in situ* hybridization of IL-17 mRNA of anti-thy1 glomerulonephritic animals on d5. Representative images showing glomerular protein expression (A) and *in situ* hybridization (B) of IL-17 mRNA (dark purple) of anti-thy1 glomerulonephritic animals on d5 after disease induction, in paraffin-embedded renal sections at x400 magnification. The black arrows indicate IL-17protein or mRNA deposition.

**Fig 6:**
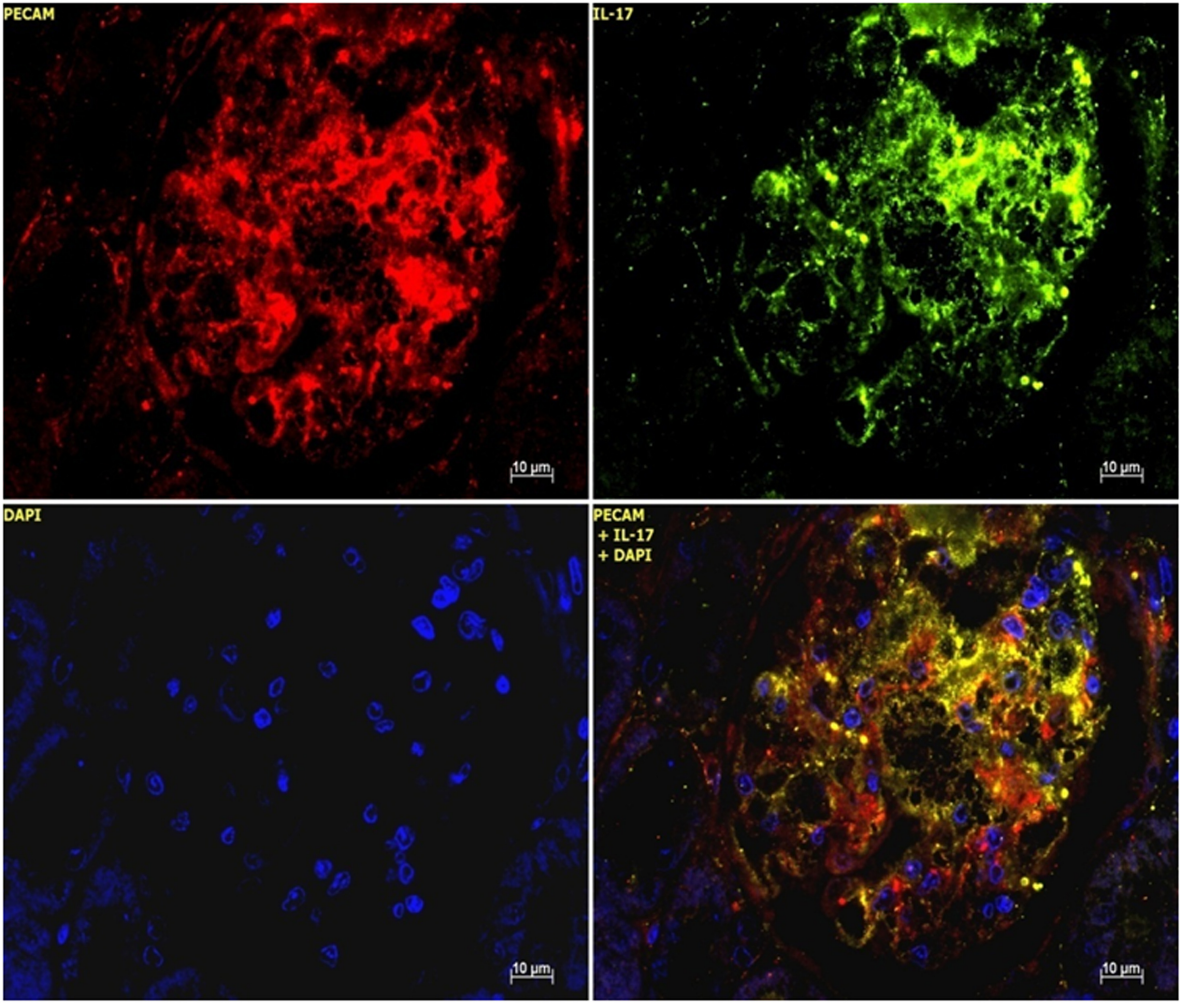
IL-17 and PECAM-1 protein expression of an anti-thy1 glomerulonephritic animal on d5. Representative images showing glomerular IL-17 and PECAM-1 protein expression in renal tissue sections of an anti-thy1 glomerulonephritic animal on d5 after disease induction, in paraffin-embedded immunostained sections at x400 magnification. IL-17 green, PCAM-1 red, nucleus blue, merge yellow.

**Fig 7:**
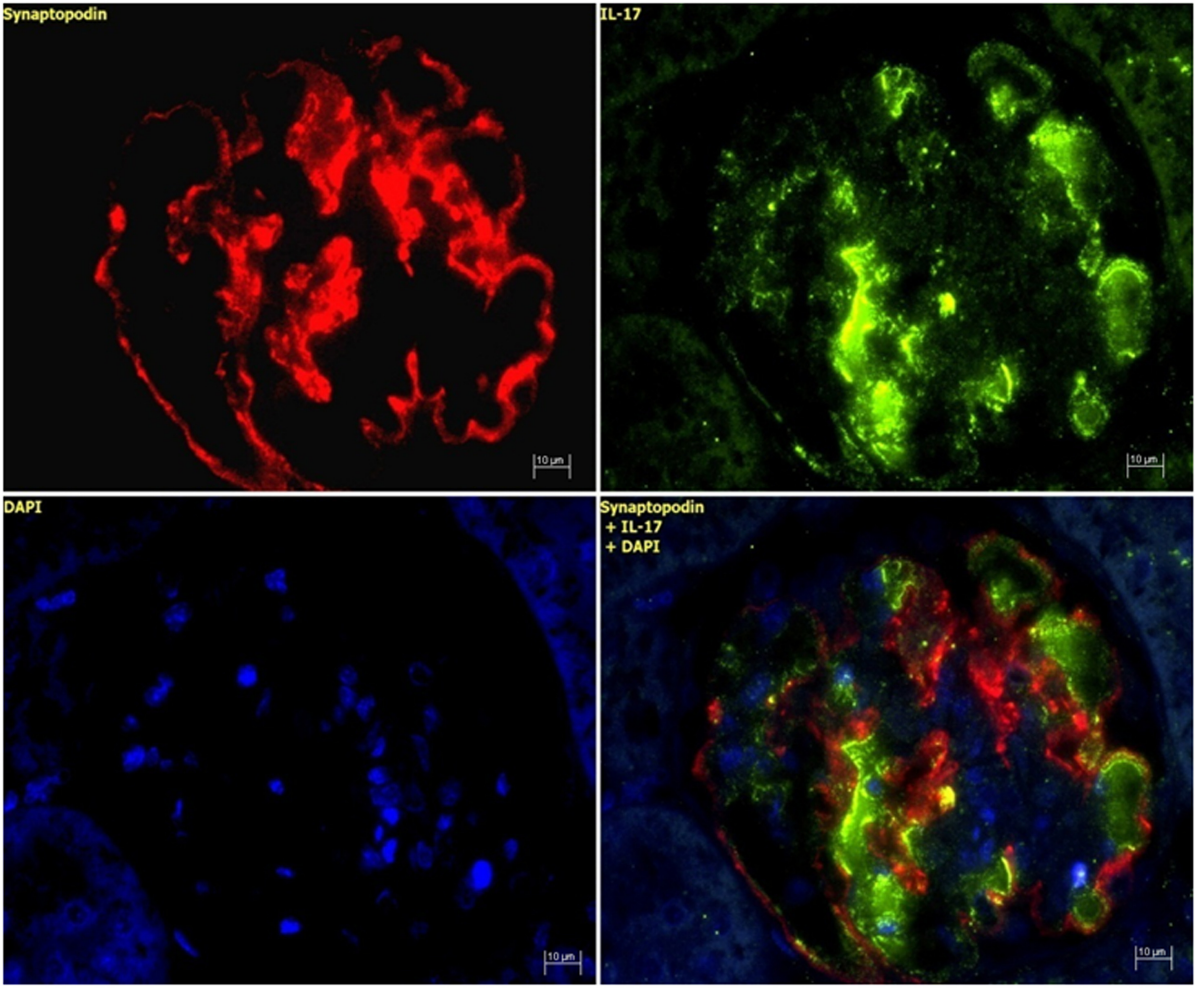
IL-17 and synaptopodin protein expression of an anti-thy1 glomerulonephritic animal on d5. Representative images showing glomerular IL-17 and synaptopodin protein expression in renal tissue sections of an anti-thy1 glomerulonephritic animal on d5 after disease induction, in paraffin-embedded immunostained sections at x400 magnification. IL-17 green, synaptopodin red, nucleus blue, merge yellow.

In line with the mRNA data, immunostaining demonstrated a considerable increase of IL-17 protein on d5 and a gradual decline thereafter (Fig 4). In order to identify the cellular source of IL-17 expression, classical APAAP immunostaining as well as *in situ* hybridization was performed. IL-17 protein and mRNA were seen to be stained in endothelial cells of d5 nephritic glomeruli, as indicated by the black arrows in Fig 5A (protein) and 5B (mRNA). This was confirmed by double staining. The endothelial cell marker PECAM-1 co-localized with IL-17 staining in glomerular cells on day 5 after induction of acute anti-thy1 glomerulonephritis, as indicated by the yellow merge in Fig 6, while there was no merge with the mesangial cell marker OX-7 or the podocyte cell marker synaptopodin (Fig 7).

### NRK-52E cells as an in vitro model to analyze TGF-β1, IL-6 and IL-17 interaction

**Fig 8:**
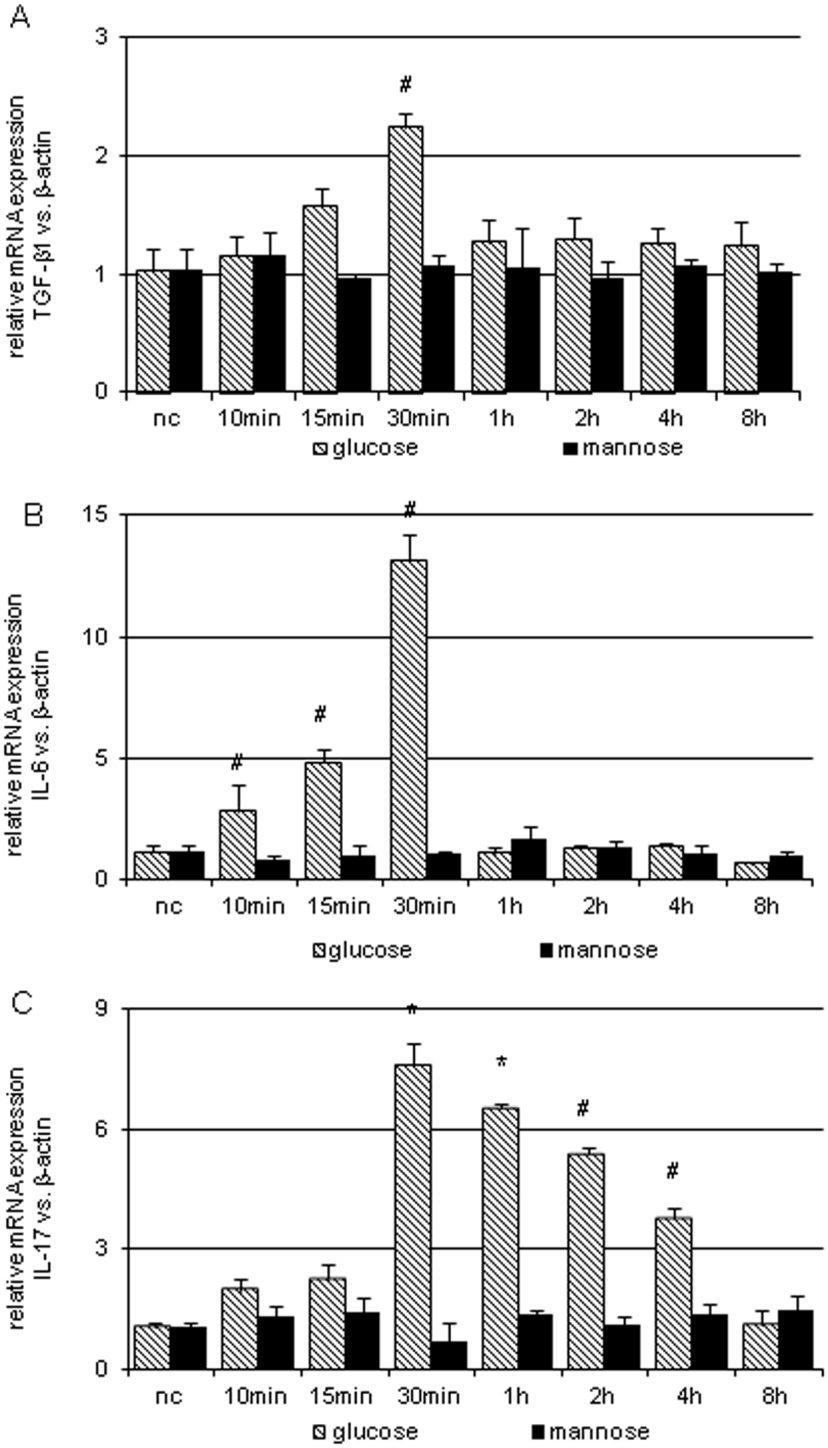
TGF-β1, IL-6 and IL-17 mRNA expression of NRK-52E after glucose stimulation. Relative TGF-β1 (A), IL-6 (B) and IL-17 (C) mRNA expression of non-stimulated NRK-52E (nc) and at different time points after stimulation by 25 mM glucose or 20 mM mannose as an osmotic control. Analysis utilized real-time PCR and was normalized to β-actin as housekeeping gene. (# p <0.05 vs. nc; * p<0.001 vs. nc).

NRK-52E cell line express low levels of TGF-β1, IL-6 and IL-17 mRNA under basal conditions (Fig 8). The exposure to 25 mM glucose (high glucose, HG) as an injury stimulus significantly enhanced simultaneously TGF-β1 by 2.2-fold (p<0.01; 30 min), IL-6 mRNA by 13.1-fold; p<0.001; 30 min) and IL-17 mRNA by 7.6-fold; (p<0.001) 30 min after stimulation. In the presence of 20 mM mannose and 5 mM glucose as a comparable osmotic control, no significant changes were found in TGF-β1, IL-6 and IL-17 mRNA expression.

### TGF-β1/ IL-6 co-administration synergistically induces IL-17 mRNA expression in NRK-52E cells

**Fig 9:**
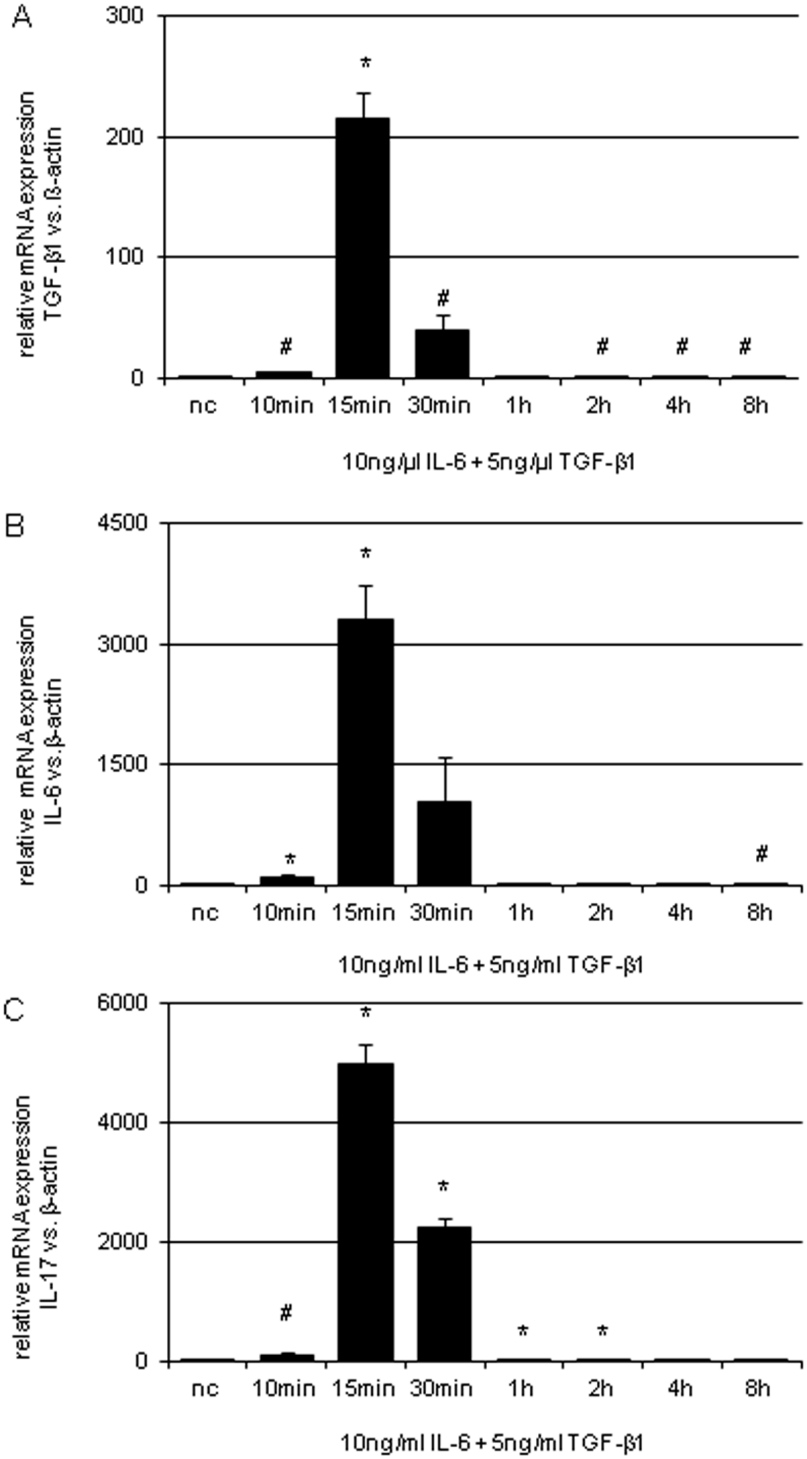
TGF-β1, IL-6 and IL-17 mRNA expression of NRK-52E after co-stimulation by TGF-β1 and IL-6. Relative TGF-β1 (A), IL-6 (B) and IL-17 (C) mRNA expression of non-stimulated NRK-52E (nc) and at different time points after co-stimulation by 5 ng/ml TGF- β1 and 10 ng/ml IL-6. Analysis utilized real-time PCR and was normalized to β-actin as housekeeping gene. (# p <0.05 vs. nc; * p<0.001 vs. nc).

**Fig 10:**
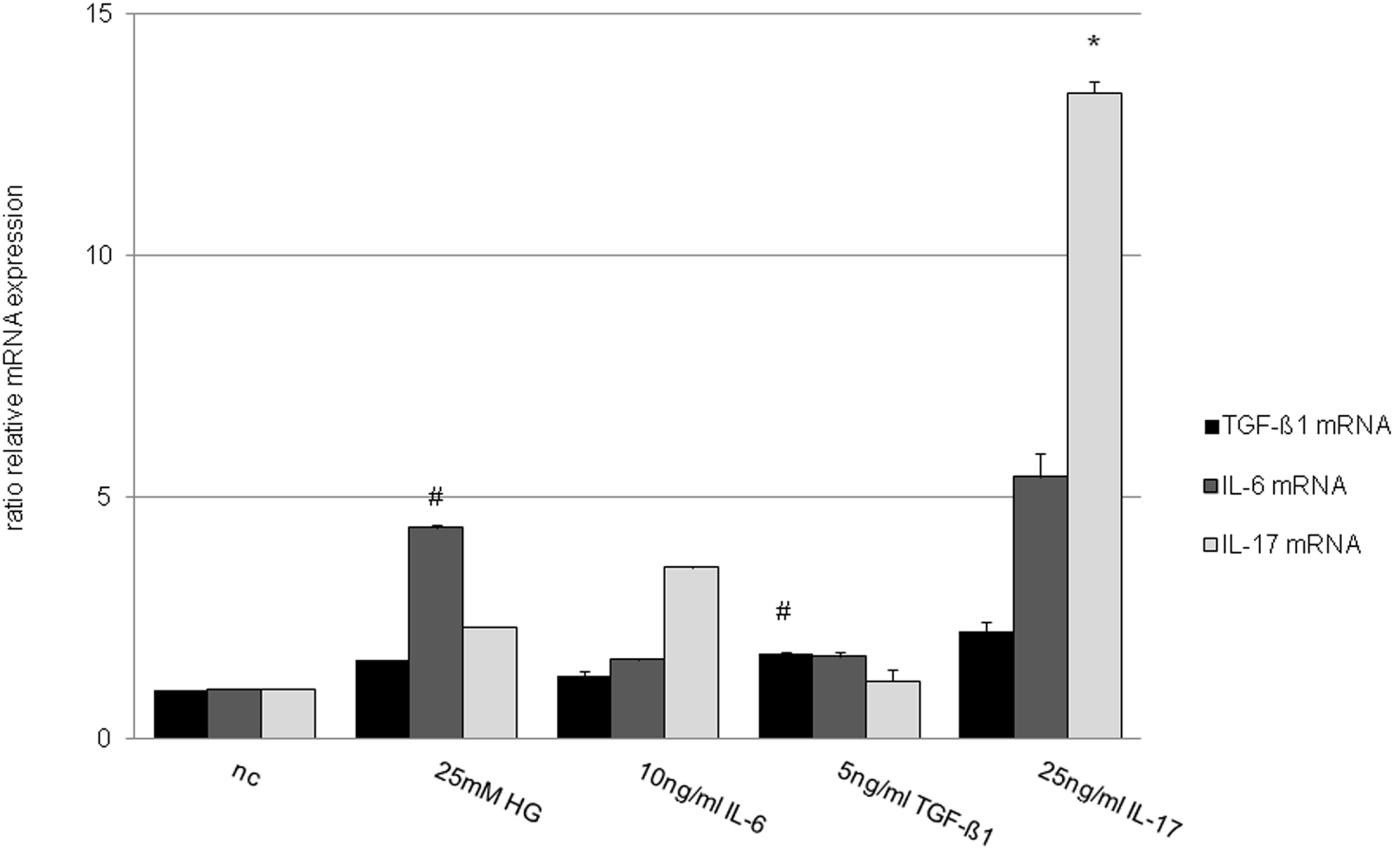
TGF-β1, IL-6 and IL-17 mRNA expression of NRK-52E after glucose, IL-6, TGF-β1 or IL-17 stimulation. TGF-β1, IL-6 and IL-17 mRNA expression in NRK-52E cells 15 min after stimulation by 25 mM glucose, 10 ng/ml IL-6, 5 ng/ml TGF-β1 or 25 ng/ml IL-17. Analysis utilized real-time PCR and was normalized to β-actin as housekeeping gene. (# p <0.05 vs. nc; * p<0.01 vs. nc).

TGF-β1 as the key mediator of fibrosis and the classic proinflammatory cytokine IL-6 are known to synergistically induce CD4^+^ cells to develop into TH17 via activation of the STAT3/RORγt pathway. To analyze this interaction in renal cells, NRK-52E cells were co-stimulated by TGF-β1 (5 ng/ml) and IL-6 (10 ng/ml). See Fig 9. Dual stimulation led to a high synergistical enhancement of IL-17 mRNA expression in NRK-52E by 4986-fold (p<0.001). In parallel, an up-regulation of TGF-β1 mRNA by 216-fold (p<0.01) and IL-6 mRNA by 3312-fold (p<0.01) in NRK-52E within 15 min was observed. In the doses employed in this study, no effective cell death was observed as analyzed by MTT vitality and BrdU proliferation testing (data not shown). The rather moderate effect of single factor expose (high glucose, TGF-β1, IL-6 and IL-17) on the mRNA expression of the corresponding cytokines is shown in Fig 10. That the synergistic action of TGF-β1/ IL-6 co-administration on IL-17 mRNA expression is at a maximum at 15 min and decreases thereafter was depicted in Fig 9.

### TGF-β receptor kinase inhibition abolishes IL-17 expression

**Fig 11:**
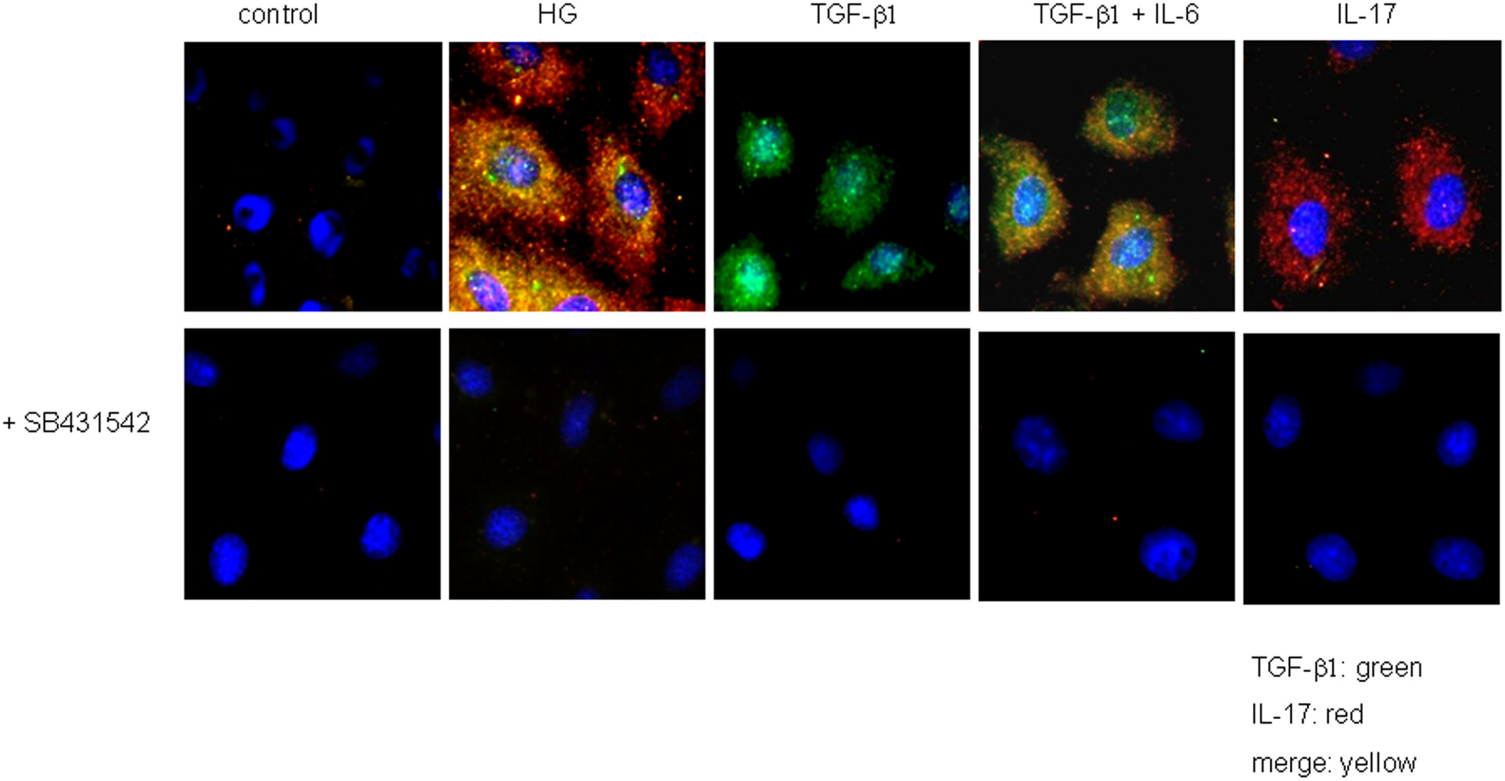
Immunofluorescence of NRK-52E after stimulation. Representative images showing IL-17 (red) and TGF-β1 (green) expression, merge (yellow), nucleus (blue) of NRK-52E cells. Row one shows untreated NRK-52E cells, NRK-52E cells stimulated by 25 mM glucose (high glucose), 5 ng/ml TGF-β1, 25 ng/ml IL-17, 5 ng/ml TGF-β1+ 10 ng/ml IL-6. Row two depicts TGF-β1 and IL-17 expression in NRK-52E after treatment by 10 μM TGF-β receptor blocker SB431542 after stimulation by 25 mM glucose (high glucose), 5 ng/ml TGF-β1, 25 ng/ml IL-17, 5ng/ml TGF-β1+ 10 ng/ml IL-6. All cells are starved 24 h for cell cycle synchronization and stimulated for 48 h at 37°C under 5% CO_2_ and then immune-stained (magnification x1000).

We next investigated TGF-β1 and IL-17 protein expression in NRK-52E cells by immunofluorescence staining. NRK-52E cells show an up-regulation in TGF-β1 and IL-17 protein expression after exposure to 25 mM glucose. Furthermore, one single stimulation with 5 ng/ml of TGF-β1 or 25 ng/ml IL-17 resulted in an auto-induction of both cytokines. Similar to the mRNA results above, a synergistic action on IL-17 protein expression was also found when the cells were co-incubated with 5 ng/ml TGF-β1 and 10 ng/ml IL-6 (Fig 11). When the cells were exposed to SB 431542 (at 10 μM a specific inhibitor of the TGF-β superfamily activin receptor-like kinase (ALK)), the expressions of both TGF-β1 and IL-17 were largely lost after stimulation using high glucose, TGF-β1 and TGF-β1/ IL-6 co-stimulation.

## Discussion

The present study documents a time-dependent expression of the new pro-inflammatory cytokine IL-17 in renal endothelial cells during the course of acute anti-thy1 glomerulonephritis. Furthermore, parallel renal cell culture stimulation and inhibition experiments suggest a synergism of TGF-β1 and IL-6 in IL-17 up-regulation, and a TGF-β1/ IL-17 network which is operative in resident renal cells.

The important role of IL-17 and TH17 cells in inflammatory processes has been recognized over the last few years. The pro-inflammatory cytokine IL-17 is a member of a new cytokine family which shows only few or even no homologies with other interleukins [37] and is expressed by activated Th17 cells, which are important mediators of autoimmune response [23,38]. Most experimental evidence to date suggests a role of IL-17 in coordinating local tissue inflammation via induced release of pro-inflammatory and granulocyte mobilizing cytokines. In 2007, Cortez et al. first found that non-immune cells such as cardiac fibroblasts are able to secrete IL-17 [39]. IL-17 receptors are expressed ubiquitously, suggesting the ability of IL-17 to operate via autocrine and paracrine mechanisms. Recently, IL-17-producing Th17 cells were identified to be involved in kidney inflammation [40,41]. Additionally, Loverre et al. showed IL-17 expression by tubular endothelial cells in renal transplant during acute antibody-mediated rejection [31]. A large number of recent studies has established a central role of the profibrotic cytokine TGF-β1 and the proinflammatory cytokine IL-6 for TH17 cell differentiation and secretion of IL-17 [26,27,42] and shown that both are markedly involved in pathogeneses of acute and chronic kidney disease. Thus, we addressed the question of a TGF- β1/ IL-6/ IL-17 network in resident renal cells. To investigate IL-17 expression in renal cells over the time course of acute glomerulonephritis, a model of acute anti-thy1 glomerulonephritis in rats was used. Due to a single OX-7 antibody injection, a temporary complement and NO-mediated mesangial cell lysis occurred [43]. The model used resembles to some extent human IgA-nephropathy [33] and shows marked acute extracellular matrix expansion. Acute anti-thy1 glomerulonephritis in the rat is characterized by an injury phase (6–48 h after induction) with mesangial cell lysis, macrophage influx, and overexpression of proinflammatory cytokines such as TNF-α and IL-1. Followed by matrix expansion phase (d5 to d12) with increased mesangial cell proliferation, extracellular matrix expansion, and micro-aneurysm in glomerular capillaries, overexpression of profibrotic cytokines (TGF-β1, activin A) and matrix proteins (collagen and fibronectin) took place. The molecular mechanism of extracellular matrix expansion is similar to the mechanism in diabetic [44] and hypertensive nephropathy [45].

The cytokine TGF-β1 is a key mediator during of extracellular matrix expansion and kidney fibrosis formation. Findings by Border and Noble with overexpression of TGF-β1 in renal fibrosis and successful use of neutralizing anti-TGF-β1 antibody demonstrated that the cytokine TGF-β1 plays as a major role in kidney fibrosis during acute anti-thy1 glomerulonephritis [5]. The present study documents a high up-regulation of TGF-β1 protein and mRNA expression with the highest peak seen on d5 (matrix expansion phase), which then declines over the following days. Furthermore, we found an up-regulation of the pro-inflammatory cytokine IL-6 on d5 of acute anti-thy1 glomerulonephritis, confirming recent experimental studies which demonstrated convincingly that IL-6 is a strong mediator of mesangial cell proliferation and matrix expansion in glomerulonephritis [46–48].

We speculate that the considerable up-regulation of IL-17 expression on d5, as seen at the mRNA and protein levels, is a result of TGF-β1 and IL-6 up-regulation. To our knowledge this is the first time a significant increase of IL-17 mRNA and protein in acute anti-thy1 glomerulonephritis has been detected. Our findings—that TGF-β1, IL-6 and IL-17 are coordinately up-regulated in the matrix expansion phase—provide strong evidence for an interaction in the genesis of acute glomerulonephritis.

We also showed by double staining analyses that the increased IL-17 expression found on day 5 is unique for renal endothelial cells. No merge in other resident renal cells (e.g. podocytes, mesangial cells) was observed. Various renal cells (mesangial cells, tubular epithelial cells, fibroblast, podocytes), non-renal cells (lymphocytes, macrophages, thrombocytes) and systemic factors like hemodynamic entities are involved in the development of acute and chronic glomerulonephritis. Due to this, today's investigations of interactions in cytokine networking have clearly become a challenging event. Therefore TGF-β1/ IL-17 interaction was examined using *in vitro* experiments, since conditions and influence of known and unknown biological parameters can be standardized. In 2007, Cortez et al. presented for the first time data which demonstrate that non-immune, non-endothelial cells, i. e. cardiac fibroblasts, have an ability to express low levels of IL-17 [39]. We choose for our *in vitro* the well established renal tubular epithelial cell line, NRK-52E. In pilot experiments, we had found reproducible IL-17 mRNA and protein expression by TGF-β and IL-6. Other groups had employed NRK-52E for pathomechanism involved in glomerular alterations [49]. Subsequently Venkatachalam et al. showed that stimulation of primary mouse cardiac fibroblasts by high glucose levels (25 mM) induces increased IL-17 expression via PI3K→Akt→ERK signaling that was abolished by PI3K inhibition via resveratrol [50]. Since high glucose concentrations can act as an unspecific injury stimulus and thus no further cytokine like TNF-α is required, we simply used a high glucose concentration as a stimulation in our investigations. Venkatachalam's finding is in a line with our investigations of time-dependent IL-17 expression increase in NRK-52E cells after a high glucose stimulation. We found a significantly increased IL-17 mRNA and protein expression in renal tubular epithelial cells by synergistic TGF-β1 and IL-6 stimulation, suggesting a regulatory connection between these. Wu and Derynck showed that glucose stimulation induces TGF-β1 expression via c-terminal phosphorylation of Smad 3, similar to activation in response to TGF-β1 and glucose induces nuclear translocation of Smad2/3 as well as PI3K→Akt→mTOR activation are blocked by SB421542 [51]. However our data indicate that there may in fact be a regulatory key mechanism in the TGF-β1/ IL-17 network of resident renal cells, since TGF-β receptor antagonism *in vitro* by selective TGF-β receptor type 1 blockade using SB431542 down-regulates IL-17 expression. We found an abolishment of IL-17 expression by SB431542 treatment after IL-17 auto-induction, induction by TGF-β1/ IL-6 co-stimulation, as well as unspecific injury stimulation by high glucose contents. In 2008, Nardelli et al. showed a similar effect in experimental destructive arthritis in mice. They found that co-treatment with anti-TGF-β1, anti-IL-6 and anti-IL-17 antibody reduced the severity of arthritis significantly more than the inhibition of single cytokines in Borellia-vaccinated and challenged mice did [52]. This underlines the imperative usage of a TGF-β RI kinase activity for these effects. This finding suggest that the key cytokines TGF-β1 and IL-6 work together with IL-17 during acute anti-thy1 glomerulonephritis and a potential therapy may aim at a concerted inhibition of these cytokines.

## Conclusions

Our study documents a time-dependent expression of the pro-inflammatory cytokine IL-17 in renal endothelial cells in acute anti-thy1 glomerulonephritis. *In vitro*, NRK-52E cells secrete IL-17 under pro-fibrotic and pro-inflammatory conditions. This result indicates that IL-17 is part of the link between inflammation and fibrosis, and is involved in the in the pathogenesis of acute glomerulonephritis.

## Supporting Information

S1 Table IL-17 Expression in the Time Course of Acute Anti-Thy1 Glomerulonephritis File Dataset. (PDF)
