# Supplementary material for: IL-17 Expression in the Time Course of Acute Anti-Thy1 Glomerulonephritis
Source: PLoS One. 2016 May 31;11(5):e0156480. doi: 10.1371/journal.pone.0156480 (PMC4886969; doi:10.1371/journal.pone.0156480)
Supplement: S1 Table — (PDF) [file pone.0156480.s001.pdf]

S1 Table. IL-17 Expression in the Time Course of Acute Anti-Thy1 Glomerulonephritis File Dataset.

aGN						
		proteinuria	matrixscore	mRNA	mRNA	mRNA
**animal**		**mg/24h**	**%**	**TGF-ß**	**IL-6**	**IL-17**
**1**	**con**	31	29,0	1,197	1,444	1,332
**2**		37	30,5	1,240	1,753	0,685
**3**		33	28,5	1,288	0,620	1,489
**4**		34	27,0	0,516	0,635	0,734
**5**		20	26,0	1,187	1,357	0,889
**6**		18	30,5	0,916	0,989	1,236
	**MW**	**29**	**29**	**1,06**	**1,13**	**1,06**
	**STABW**	**7,860**	**1,819**	**0,295**	**0,461**	**0,336**
	**SEM**	**3,209**	**0,742**	**0,121**	**0,188**	**0,137**
**7**	**24h**	37	23,0	0,804	0,519	1,292
**8**		36	20,5	1,261	0,689	0,642
**9**		46	21,5	1,121	0,427	1,676
**10**		38	26,0	0,889	1,012	0,631
**11**		43	22,0	1,070	0,957	0,667
**12**		38	20,5	0,967	0,537	0,710
	**MW**	**40**	**22**	**1,02**	**0,69**	**0,94**
	**STABW**	**3,933**	**2,068**	**0,166**	**0,244**	**0,442**
	**SEM**	**1,606**	**0,844**	**0,068**	**0,100**	**0,181**
	**TT vs con**	**0,017**	**0,000**	**0,788**	**0,073**	**0,595**
	**TT vs d5**	**0,002**	**0,000**	**0,048**	**0,003**	**0,003**
**13**	**d5**	60	85,5	2,313	14,929	5,837
**14**		121	80,0	4,302	58,892	2,091
**15**		99	89,5	1,098	48,840	7,219
**16**		115	70,5	1,253	21,407	5,712
**17**		145	80,5	2,037	31,810	8,840
**18**		136	85,5	2,454	40,227	9,331
	**MW**	**113**	**82**	**2,24**	**36,02**	**6,50**
	**STABW**	**30,325**	**6,621**	**1,151**	**16,621**	**2,627**
	**SEM**	**12,380**	**2,703**	**0,470**	**6,786**	**1,072**
	**TT vs con**	**0,001**	**0,000**	**0,049**	**0,004**	**0,004**
	**TT vs d5**					
**19**	**d10**	93	75,5	0,041	0,228	5,112
**20**		40	63,5	1,057	0,609	1,833
**21**		116	82,0	0,205	1,003	5,441
**22**		59	85,5	0,012	0,193	5,896
**23**		44	74,5	0,426	0,427	4,518
**24**		142	85,0	0,226	0,024	4,447
	**MW**	**82**	**78**	**0,33**	**0,41**	**4,54**
	**STABW**	**41,680**	**8,359**	**0,387**	**0,352**	**1,437**
	**SEM**	**17,016**	**3,412**	**0,158**	**0,144**	**0,586**
	**TT vs con**	**0,025**	**0,000**	**0,005**	**0,014**	**0,002**
	**TT vs d5**	**0,182**	**0,353**	**0,008**	**0,003**	**0,148**
**25**	**d15**	113	64,5	0,260	0,322	1,858
**26**		58	59,0	0,325	0,781	3,326
**27**		34	51,0	0,088	1,214	3,926
**28**		50	53,0	0,087	1,235	2,876
**29**		37	49,5	0,153	0,630	7,433
**30**		86	69,0	0,198	0,050	4,095
	**MW**	**63**	**58**	**0,19**	**0,71**	**3,92**
	**STABW**	**30,846**	**7,872**	**0,096**	**0,475**	**1,901**
	**SEM**	**12,593**	**3,214**	**0,039**	**0,194**	**0,776**
	**TT vs con**	**0,040**	**0,000**	**0,000**	**0,145**	**0,014**
	**TT vs d5**	**0,019**	**0,000**	**0,007**	**0,003**	**0,082**
**19**	**d20**	63	40,5	0,529	0,132	2,936
**20**		29	54,5	0,412	0,642	0,545
**21**		61	55,0	0,171	0,213	3,901
**22**		48	45,5	0,004	0,000	0,263
**23**		40	36,0	0,229	0,227	1,725
**24**		42	34,0	0,309	0,268	2,040
	**MW**	**47**	**44**	**0,28**	**0,25**	**1,90**
	**STABW**	**12,852**	**9,048**	**0,185**	**0,215**	**1,389**
	**SEM**	**5,247**	**3,694**	**0,075**	**0,088**	**0,567**
	**TT vs con**	**0,017**	**0,007**	**0,000**	**0,004**	**0,203**
	**TT vs d5**	**0,002**	**0,000**	**0,008**	**0,003**	**0,006**

NRK52E

	5ng/ml TGF-ß + 10ng/ml IL-6 relative mRNA expression			HG relative mRNA expression			HM relative mRNA expression		
	TGF-ß	IL-6	IL-17	TGF-ß	IL-6	IL-17	TGF-ß	IL-6	IL-17
nc	1,007	1,316	0,601	1,429	1,537	1,526	1,505	1,873	1,479
nc	1,479	0,878	1,635	1,240	1,035	0,683	1,066	1,261	0,662
nc	0,859	1,341	0,596	1,057	0,483	1,393	0,909	0,588	1,039
nc	0,768	0,885	1,382	0,409	1,301	0,599	0,643	0,719	0,983
**MW**	**1,03**	**1,10**	**1,05**	**1,03**	**1,09**	**1,05**	**1,03**	**1,11**	**1,04**
**STABW**	**0,316**	**0,258**	**0,535**	**0,443**	**0,453**	**0,477**	**0,361**	**0,586**	**0,336**
**SEM**	**0,158**	**0,129**	**0,268**	**0,222**	**0,227**	**0,239**	**0,181**	**0,293**	**0,168**
10min	7,413	111,921	121,347	1,268	2,990	1,731	1,151	1,688	1,834
10min	6,298	9,801	63,838	0,902	2,732	2,828	1,526	1,634	1,253
10min	4,874	114,272	184,780	1,101	2,014	1,021	1,044	1,697	0,886
10min	3,074	134,333	84,624	1,125	2,569	2,235	0,887	1,306	1,326
**MW**	**5,41**	**92,58**	**113,65**	**1,10**	**2,58**	**1,95**	**1,15**	**1,58**	**1,32**
**STABW**	**1,875**	**56,096**	**53,049**	**0,150**	**0,413**	**0,767**	**0,272**	**0,186**	**0,390**
**SEM**	**0,937**	**28,048**	**26,524**	**0,075**	**0,206**	**0,383**	**0,136**	**0,093**	**0,195**
**TT vs nc**	**0,017**	**0,047**	**0,024**	**0,794**	**0,003**	**0,102**	**0,613**	**0,208**	**0,314**
15min	202,953	2909,052	5478,885	1,192	5,368	2,310	0,912	1,512	1,406
15min	182,278	4123,537	5606,948	1,985	5,426	0,979	1,021	1,740	0,809
15min	276,282	2643,072	4649,952	1,246	4,084	1,959	0,871	2,107	1,400
15min	200,854	3573,198	4210,181	1,814	4,570	3,766	1,089	1,445	2,035
**MW**	**215,59**	**3312,21**	**4986,49**	**1,56**	**4,86**	**2,25**	**0,97**	**1,70**	**1,41**
**STABW**	**41,513**	**667,495**	**669,162**	**0,400**	**0,649**	**1,155**	**0,100**	**0,298**	**0,500**
**SEM**	**20,757**	**333,747**	**334,581**	**0,200**	**0,325**	**0,577**	**0,050**	**0,149**	**0,250**
**TT vs nc**	**0,002**	**0,002**	**0,001**	**0,129**	**0,000**	**0,126**	**0,776**	**0,139**	**0,270**
30min	23,752	204,081	1737,744	2,549	15,102	7,384	0,930	2,645	0,713
30min	60,338	1803,187	2544,206	1,959	12,788	7,144	1,119	2,235	0,338
30min	17,815	467,774	2292,967	2,173	11,525	8,437	1,114	2,692	1,653
30min	61,606	1640,212	2346,563	2,217	12,986	7,602	1,136	2,390	0,070
**MW**	**40,88**	**1028,81**	**2230,37**	**2,22**	**13,10**	**7,64**	**1,07**	**2,49**	**0,69**
**STABW**	**23,335**	**810,023**	**345,732**	**0,244**	**1,483**	**0,562**	**0,097**	**0,216**	**0,692**
**SEM**	**11,667**	**405,011**	**172,866**	**0,122**	**0,742**	**0,281**	**0,049**	**0,108**	**0,346**
**TT vs nc**	**0,042**	**0,085**	**0,001**	**0,006**	**0,000**	**0,000**	**0,827**	**0,013**	**0,414**
1h	0,653	0,683	7,343	1,071	1,000	6,143	0,652	0,629	1,210
1h	2,704	0,628	8,834	1,102	1,000	6,021	1,052	1,387	1,227
1h	0,895	0,624	7,925	1,240	1,000	6,816	1,549	0,635	1,641
1h	1,419	0,911	8,454	1,680	2,290	7,013	0,941	1,293	1,359
**MW**	**1,42**	**0,71**	**8,14**	**1,27**	**1,32**	**6,50**	**1,05**	**0,99**	**1,36**
**STABW**	**0,915**	**0,136**	**0,649**	**0,281**	**0,645**	**0,490**	**0,374**	**0,410**	**0,200**
**SEM**	**0,458**	**0,068**	**0,324**	**0,141**	**0,323**	**0,245**	**0,187**	**0,205**	**0,100**
**TT vs nc**	**0,470**	**0,047**	**0,000**	**0,402**	**0,578**	**0,000**	**0,949**	**0,741**	**0,166**
2h	0,371	5,476	18,417	1,540	1,570	6,692	0,903	0,832	1,116
2h	0,243	5,501	32,968	1,123	0,966	4,199	0,980	1,702	0,798
2h	0,183	1,681	18,375	1,248	1,157	4,993	1,146	1,621	1,429
2h	0,221	1,257	35,498	1,256	1,347	5,583	0,870	0,697	1,129
**MW**	**0,25**	**3,48**	**26,31**	**1,29**	**1,26**	**5,37**	**0,97**	**1,21**	**1,12**
**STABW**	**0,082**	**2,327**	**9,202**	**0,177**	**0,191**	**0,695**	**0,139**	**0,559**	**0,315**
**SEM**	**0,041**	**1,163**	**4,601**	**0,088**	**0,095**	**0,347**	**0,070**	**0,279**	**0,158**
**TT vs nc**	**0,013**	**0,133**	**0,012**	**0,341**	**0,801**	**0,002**	**0,879**	**0,623**	**0,767**
4h	3,193	0,707	0,285	1,246	1,728	3,310	0,892	2,955	1,352
4h	1,564	0,831	3,052	1,369	1,376	3,811	1,250	1,270	1,333
4h	2,694	0,882	0,267	1,267	1,266	5,134	0,903	1,695	1,847
4h	2,329	1,009	2,975	1,070	1,214	2,713	1,240	2,772	0,898
**MW**	**2,45**	**0,86**	**1,64**	**1,24**	**1,40**	**3,74**	**1,07**	**2,17**	**1,36**
**STABW**	**0,686**	**0,125**	**1,581**	**0,124**	**0,082**	**1,031**	**0,201**	**0,819**	**0,388**
**SEM**	**0,343**	**0,063**	**0,790**	**0,062**	**0,041**	**0,515**	**0,100**	**0,410**	**0,194**
**TT vs nc**	**0,018**	**0,154**	**0,521**	**0,432**	**0,455**	**0,008**	**0,852**	**0,084**	**0,264**
8h	1,414	0,524	1,231	0,881	0,611	0,601	0,812	1,622	1,371
8h	1,945	0,628	0,120	1,647	0,758	0,801	1,089	2,740	1,169
8h	1,682	0,639	1,074	1,256	0,483	1,840	0,920	2,805	1,003
8h	1,315	0,596	0,080	1,269	0,809	1,093	1,240	2,986	1,952
**MW**	**1,59**	**0,60**	**0,63**	**1,26**	**0,67**	**1,08**	**1,02**	**2,54**	**1,37**
**STABW**	**0,284**	**0,052**	**0,611**	**0,313**	**0,148**	**0,543**	**0,188**	**0,620**	**0,414**
**SEM**	**0,142**	**0,026**	**0,306**	**0,156**	**0,074**	**0,272**	**0,094**	**0,310**	**0,207**
**TT vs nc**	**0,039**	**0,027**	**0,334**	**0,433**	**0,157**	**0,929**	**0,943**	**0,016**	**0,260**

relative mRNA expression			
	TGF-ß1	IL-6	IL-17
nc	1,037	1,236	0,966
	1,164	0,807	1,395
	0,999	1,204	0,885
**MW**	**1,066**	**1,082**	**1,082**
**STABW**	**0,086**	**0,239**	**0,274**
**SEM**	**0,050**	**0,138**	**0,158**
25mM HG	1,625	4,347	2,297
	1,600	4,452	2,346
	1,639	4,307	2,286
**MW**	**1,621**	**4,369**	**2,310**
**STABW**	**0,020**	**0,075**	**0,032**
**SEM**	**0,011**	**0,043**	**0,018**
**TT vs nc**	**0,006**	**0,001**	**0,015**
10ng/ml IL-6	1,302	1,639	3,598
	1,127	1,683	3,462
	1,421	1,590	3,549
**MW**	**1,283**	**1,637**	**3,536**
**STABW**	**0,148**	**0,047**	**0,069**
**SEM**	**0,085**	**0,027**	**0,040**
**TT vs nc**	**0,110**	**0,052**	**0,003**
5ng/ml TGF-ß	1,777	1,636	1,119
	1,643	1,869	0,993
	1,802	1,591	1,456
**MW**	**1,741**	**1,699**	**1,189**
**STABW**	**0,086**	**0,149**	**0,239**
**SEM**	**0,049**	**0,086**	**0,138**
**TT vs nc**	**0,001**	**0,027**	**0,637**
25ng/ml IL-17	2,286	5,957	13,387
	2,367	4,452	13,769
	2,016	5,821	12,922
**MW**	**2,223**	**5,410**	**13,359**
**STABW**	**0,184**	**0,832**	**0,424**
**SEM**	**0,106**	**0,481**	**0,245**
**TT vs nc**	**0,003**	**0,008**	**0,000**
